# Synthesis of Low-Defect Iron-Based Prussian Blue with Low Water Content for High-Stability Sodium-Ion Batteries

**DOI:** 10.3390/ma18071455

**Published:** 2025-03-25

**Authors:** Zhaoyue Li, Shenglin Zhong, Bingcheng Zhou, Denglian Chen, Zehai Qiu, Rui Zhang, Ruijuan Zheng, Chenhao Zhao, Jiangcong Zhou

**Affiliations:** 1 Key Laboratory of Nonferrous Materials and New Processing Technology, Ministry of Education, Guangxi Key Laboratory of Optical and Electronic Materials and Devices, College of Materials Science and Engineering, Guilin University of Technology, Guilin 541004, China; zhaoyueli0823@163.com (Z.L.); denglianchen02@163.com (D.C.); 2 College of Chemistry and Materials Science, Longyan University, Longyan 364012, China; 18190315877@163.com (B.Z.); 81993016@lyun.edu.cn (Z.Q.); juanzr1234@163.com (R.Z.); zhaochenhao123456@163.com (C.Z.)

**Keywords:** sodium-ion batteries, Prussian blue analogs, low water content, iron-based materials, electrochemical performance

## Abstract

This study proposes an innovative two-step synthesis strategy to significantly enhance the performance of sodium-ion batteries by developing low-defect, low water content iron-based Prussian blue (PB) materials. Addressing the limitations of traditional co-precipitation methods—such as rapid reaction rates leading to excessive crystal defects and interstitial water content—the research team introduced a synergistic approach combining non-aqueous phase precursor synthesis and controlled water-concentration secondary crystallization. The process involves preparing a PB precursor in a glycerol system, followed by secondary crystallization in a water-/ethanol-mixed solvent with a precisely regulated water content, achieving the dual objectives of water content reduction and crystal morphology optimization. Systematic characterization revealed that water concentration during secondary synthesis critically influences the material’s crystal structure, morphological features, and water content. The optimized PB50-24 material exhibited a highly regular cubic morphology with a sodium content of 9.2% and a remarkably low interstitial water content of 2.1%. Electrochemical tests demonstrated outstanding performance—an initial charge–discharge capacity of 120 mAh g^−1^ at a 1C rate, the retention of 105 mAh g^−1^ after 100 cycles, and a high rate capability of 86 mAh g^−1^ at 10C, representing significant improvements in cycling stability and rate performance over conventional methods. This work not only establishes a cost-effective, scalable synthesis pathway for Prussian blue materials but also provides theoretical guidance for developing other metal-based Prussian blue analogs, offering substantial value for advancing the industrial application of sodium-ion batteries in next-generation energy storage systems.

## 1. Introduction

Sodium reserves are abundant, and sodium, which belongs to the same main group as lithium, is significantly less expensive. Sodium-ion batteries (SIBs) share similar charging and discharging principles with lithium-ion batteries (LIBs) [1,2,3,4], making them one of the most promising candidates to replace LIBs in new energy storage applications [5,6,7]. Currently, the anodes of SIBs predominantly employ graphite materials [8], while the cathode materials are more diverse and play a critical role in enhancing electrochemical performance [9]. There are three main types of cathode materials [10] under investigation—layered oxides, polyanionic compounds, and Prussian blue analogs (PBAs) [11]. Prussian blue analogs (M_x_M’_1−x_ [Fe (CN)_6_], where M and M’ are transition metal ions and x represents the proportion of these metal ions [12]) are materials with a crystal structure akin to Prussian blue (Fe_4_[Fe (CN)_6_]_3_·xH_2_O). Typically composed of transition metal ions and cyanide compounds, they form a three-dimensional framework [13]. The crystal structure of PBAs facilitates the efficient insertion and extraction of metal ions, endowing them with superior electrochemical performance. Due to their tunable structural properties, PBAs are widely used in sodium-ion batteries and other energy storage technologies. Prussian blue analogs are considered the most promising cathode materials for large-scale industrialization due to their unique structural characteristics, low-temperature synthesis via co-precipitation, scalability, and stable voltage platforms during charging and discharging [14].

However, the traditional co-precipitation method, which involves direct synthesis in an aqueous phase, often results in excessively fast reaction rates [15]. This leads to defects during crystal growth, the incorporation of water molecules into the crystal lattice, and increased water content [16]. These water molecules not only occupy Na^+^ sites but also compete with Na^+^ for interstitial spaces, increasing the migration energy barrier for Na^+^ and hindering its diffusion within the lattice, thereby reducing the material’s capacity utilization [17]. Furthermore, during charging and discharging, water molecules in the lattice can migrate to the organic electrolyte, triggering electrochemical decomposition side reactions and posing significant safety risks [18]. Therefore, reducing water content and synthesizing Prussian blue crystals with regular morphology and appropriate size [19] are crucial for improving their electrochemical performance [20].

To address these challenges, researchers have explored various strategies to reduce water content [21] and optimize synthesis methods [22,23] to control crystal morphology and size. For instance, post-synthesis treatments such as high-temperature processing can effectively remove water molecules, but excessive heat may destabilize the crystal structure, leading to collapse [24,25]. A one-step presodiation and dehydration strategy for PBA nanostructures was employed to synthesize high-performance Prussian blue [26]. By using sodium benzophenone, PBA electrodes with a low water content and a high sodium content were prepared, eliminating the need for secondary drying [27]. Additionally, introducing chelating agents and Na^+^ ions during synthesis can slow down the reaction rate and increase Na^+^ content in the crystals, thereby reducing water incorporation into defects [28]. Recently, non-aqueous solvents such as ethanol have been used as reaction media to synthesize PBAs with very low water contents [29]. However, methods like microwave-assisted synthesis are often more expensive than conventional co-precipitation. While these improved synthesis and treatment methods can effectively reduce water content to some extent, they often face trade-offs. For example, slowing down the reaction rate by adding chelating agents can minimize defects, but an excessively low water content may compromise the structural stability of the crystals. Therefore, investigating the influence of water concentration during synthesis on crystal morphology, size, and water content is of great significance.

In this study, we explore the effect of water concentration on the secondary treatment of non-aqueous synthesized precursors. We propose a two-step approach as follows: first, synthesizing the precursor in a non-aqueous solvent, followed by secondary treatment with controlled water/ethanol concentrations. This method allows for the synthesis of Prussian blue crystals with tunable grain sizes. We demonstrate that the precursor synthesized in a non-aqueous solvent is not a fully formed Prussian blue crystal but rather an intermediate, and the water concentration during secondary treatment significantly influences grain size. Compared to crystals synthesized directly in an aqueous system, the secondary-treated precursor exhibits a lower water content and improved electrochemical performance. This approach, involving non-aqueous precursor synthesis followed by water-mediated secondary treatment, can also be extended to the synthesis of non-iron-based or mixed-metal Prussian blue analogs, offering valuable insights for the development of high-performance PBA materials.

## 2. Experimental

### 2.1. Materials Synthesis

The chemicals sodium citrate, sodium ferrocyanide, ferrous sulfate, and glycerol were purchased from Macklin Reagents (Shanghai Macklin, Shanghai, China) with their purity of AR analytical grade. The anhydrous ethanol was from Xilong Reagents (Xilong Scientific, Shantou, China) with a purity of ≥99.7%, and the water was ultrapure H_2_O prepared in the laboratory. According to a previous report [30], sodium citrate, sodium ferrocyanide, and ferrous sulfate are sequentially added to glycerol. The mixture is then stirred and reacted at 140 °C under a nitrogen atmosphere (20–25 mL/min). The reaction is maintained for 12 h. After the reaction is complete, the temperature is reduced to 50 °C. The reaction mixture is subsequently washed with anhydrous ethanol, followed by centrifugation to isolate the white precipitate. The white precipitate is then transferred to a solution with a specific water/ethanol ratio and stirred at 25 °C under nitrogen gas. The reaction duration is either 12 or 24 h. After the reaction, the mixture is centrifuged to collect the precipitate, which is then dried in a drying oven at 70 °C to obtain the final product. The samples were named based on the water concentration and reaction time, resulting in PB0-12, PB10-12, PB50-12, PB100-12, and PB50-24. The control sample was synthesized in the aqueous phase and is named PB-12.

### 2.2. Electrode Preparation

The cathode electrode consists of 70% active material (70 mg), 20% Ketjen Black (KB), and 10% polyvinylidene fluoride (PVDF) as the binder, along with an appropriate amount of N-methyl-2-pyrrolidone (NMP) (1.2 mL1.25 mL) [31]. These materials are thoroughly mixed into a slurry using a mortar and pestle. The slurry was then uniformly coated onto aluminum foil using a scraper (150 µm), and the coated foil was dried in a vacuum oven at 80 °C for 12 h. After drying, the electrode sheet was punched into 12 mm small disks. The coin cells were assembled in an argon atmosphere glove box, with the active electrode as the positive electrode and metallic sodium as the negative electrode. Whatman glass fiber was used as the separator, and the electrolyte used in our study consists of NaClO₄ salt dissolved in a 1:1 mixture of ethylene carbonate (EC) and propylene carbonate (PC), with 5% vinylene carbonate (FEC) added as an additive. EC primarily enhances solubility and the formation of the SEI film, PC improves the battery’s low-temperature performance, and FEC increases stability under high-voltage and high-temperature conditions. The optimized ratio of these solvents ensures superior electrolyte performance, thereby enhancing the overall electrochemical performance of the battery.

### 2.3. Materials Characterization

X-ray diffraction (XRD, */X’Pert3Powder, PANalytical B.V., Almelo, The Netherland) data were obtained using Cu Kα radiation (λ = 1.5418 Å) with a scan rate of 10°/minute over a 2θ range from 5° to 80° [32]. The elemental composition and oxidation states of the samples were determined using X-ray photoelectron spectroscopy (XPS, Thermo Fisher Nexsa, Thermo Fisher Scientific, Waltham, MA, USA). The surface morphology of the crystals was examined by scanning electron microscopy (SEM, Zeiss Sigma 300, Carl Zeiss AG, Oberkochen, DEU) and transmission electron microscope (TEM, Talos F200i, Thermo Fisher Scientific, Waltham, MA, USA). The Na and Fe content in the samples was analyzed using inductively coupled plasma atomic emission spectroscopy (ICP-AES, Varian ICP-OES 720, Agilent Technologies, Santa Clara, CA, USA). Thermogravimetric (TG) analysis was performed using a simultaneous thermal analyzer (STA449F3, NETZSCH, Selb, DEU) with a temperature range of 40–500 °C, a heating rate of 10 °C min^−1^, and a nitrogen atmosphere.. The TriStar II Plus micropore analyzer (Micromeritics Instrument Corporation, Norcross, GA, USA) was employed to test the Nitrogen adsorption–desorption isotherms and calculate the BET surface areas.

### 2.4. Electrochemical Measurements

The coin cells were assembled in a glove box (with water and oxygen content < 0.1 ppm). Metallic sodium was used as the negative electrode in the CR2025 coin cells. After assembly, the cells were subjected to constant current charge–discharge and rate capability testing at room temperature (25 °C) using a LAND CT3002A (Wuhan LAND Electronics, Wuhan, China) system. Cyclic voltammetry (CV) tests were conducted using an electrochemical workstation (CHI660E, CH Instruments, Austin, TX, USA) at a scan rate of 0.1 mV s^−1^. The EIS (Modulab XM, Solartron Analytical, Berwyn, UK) was measured between 0.01 Hz and 100 kHz and at the open circuit voltage of the cells.

## 3. Results and Discussion

**Figure 1:**
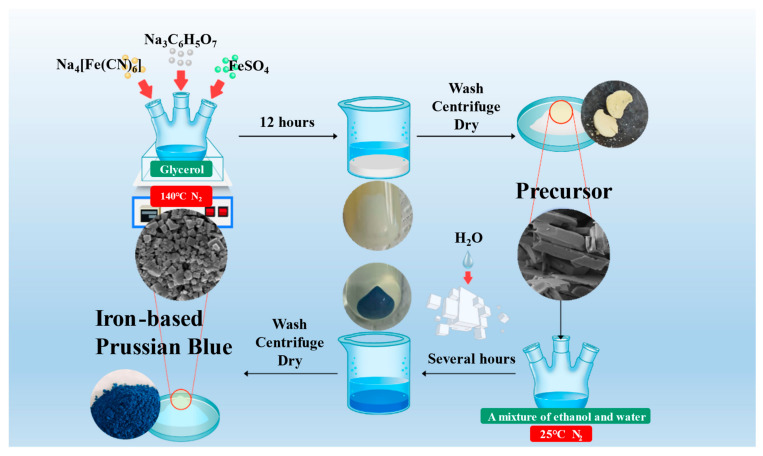
Schematic diagram of a two-step synthesis process for iron-based Prussian blue.

The synthesis diagram in Figure 1 provides a detailed description of a two-step synthesis process for iron-based Prussian blue materials. Initially, under a nitrogen atmosphere, sodium citrate, ferrous sulfate, and glycerol are added to a three-neck flask and react at 140 °C for 12 h. Subsequently, the white precursor was obtained through washing, centrifugation, and drying. Then, this precursor was placed in a solution with a specific water-to-ethanol ratio and reacted for several hours at 25 °C under a nitrogen environment for secondary crystallization. During the secondary crystallization, the precursor structure gradually transformed into the standard Prussian blue crystal structure. After the reaction was complete, the final iron-based Prussian blue material, which is blue and well-formed, was obtained through additional washing, centrifugation, oxidation, and drying steps. Throughout the entire synthesis process, the concentration of water in the water–ethanol mixture was controlled to optimize the morphology, size, and water content of the Prussian blue crystals, thereby enhancing their electrochemical performance in sodium-ion batteries.

**Figure 2:**
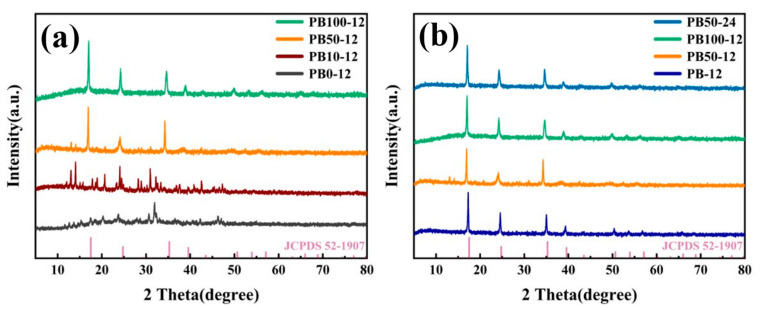
XRD patterns of Prussian blue prepared with different water contents (**a**) and reaction times (**b**).

To gain insight into the impact of varying water concentrations on the final sample’s crystalline structure, we conducted XRD analysis on the synthesized samples, with the results depicted in Figure 2. Figure 2a illustrates the XRD patterns of the samples synthesized under different water concentrations during the secondary crystallization process. PB0-12, serving as the precursor, exhibits characteristic diffraction peaks that are distinct from those of Prussian blue crystals. As the water concentration increases during secondary synthesis, the diffraction peaks of the final sample begin to resemble those of standard Prussian blue crystals when the water concentration reaches 50%, although minor impurity peaks are still present. Upon increasing the water concentration to 100%, the diffraction peaks align perfectly with those of standard Prussian blue crystals. In Figure 2b, a comparison between PB50-12 and PB50-24 reveals that extending the reaction time brings the sample’s diffraction peaks into correspondence with those of standard Prussian blue crystals. This is attributed to the longer reaction time allowing for more extensive interaction among water molecules, thereby facilitating the transformation of the crystal structure into standard Prussian blue. The data indicate that the precursor itself does not possess the crystal structure of Prussian blue. After secondary treatment, the crystal structure of the sample gradually evolves into that of Prussian blue as the water concentration increases. Under certain water concentrations, prolonging the secondary synthesis time can also completely convert non-standard Prussian blue crystals into standard Prussian blue crystals.

**Figure 3:**
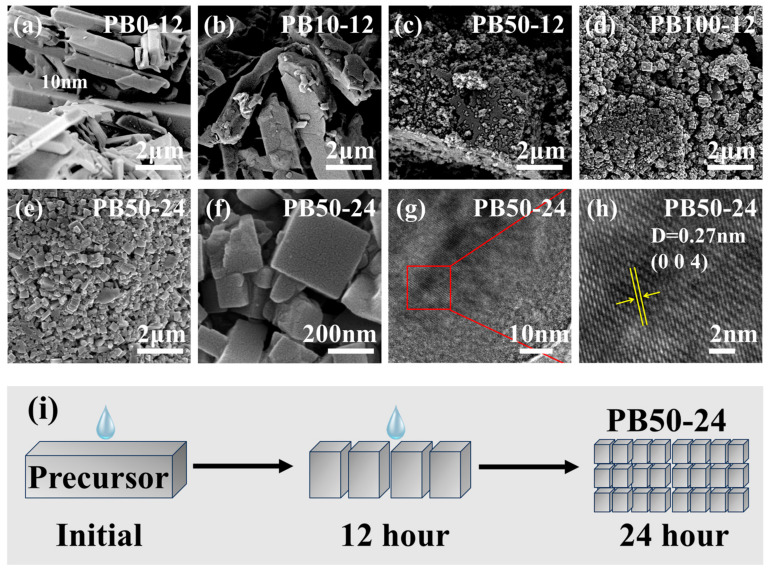
SEM images of (**a**) PB0-12, (**b**) PB10-12, (**c**) PB50-12, (**d**) PB100-12, (**e**,**f**) PB50-24, (**g**,**h**) TEM images of PB50-24, and (**i**) the schematic diagram of the synthesis mechanism for the transformation process of the iron-based Prussian blue material PB50-24.

To verify the impact of water molecules on crystal transformation, we utilized SEM to observe the microstructure of iron-based Prussian blue materials synthesized under various water concentrations and reaction times. The SEM images revealed that under anhydrous conditions (Figure 3a), the precursor consisted of large rod-like crystals. In the sample synthesized at 10% water concentration for 12 h (Figure 3b), the crystal size remained almost unchanged, but a small number of fine grains formed. At a 50% water concentration (Figure 3c), the crystal morphology underwent significant changes, with the original large rod-like crystals gradually transforming into many smaller crystals, although some larger crystals still persisted. At a 100% water concentration (Figure 3d), the crystals completely transformed into the standard cubic Prussian blue structure. When the secondary synthesis time was extended to 24 h at a 50% water concentration (Figure 3e,f), the microstructure of the sample also transformed into the standard cubic Prussian blue structure. Based on the high-resolution transmission electron microscopy images (Figure 3g,h), the clear lattice fringes show a lattice spacing of 0.27 nm, which is consistent with the (004) lattice distance. These results indicate that, as the water concentration increases and the reaction time is prolonged, the morphology and size of the crystals change, with the crystal surface becoming more regular, the size decreasing, and the crystal morphology becoming more uniform. This suggests that the transformation of the sample from the precursor to a low-defect standard Prussian blue crystal is caused by the effect of water molecules on the crystallization dynamics. The SEM and TEM images further reveal the significant influence of water concentration and reaction time on the morphology and size of Prussian blue crystals and how these conditions promote the transformation of crystals into the standard Prussian blue structure.

The schematic diagram of the synthesis mechanism in Figure 3i illustrates the transformation process of the iron-based Prussian blue material PB50-24, which is divided into three stages as follows: the initial stage, where the precursor exists as a larger, irregular block structure synthesized under anhydrous conditions; the 12 h stage, where the precursor begins to transform under the influence of water molecules, with the crystal structure starting to form but not yet fully converted into Prussian blue crystals; and the 24 h stage, where the crystal structure further develops with the extension of the reaction time, ultimately forming a regular cubic shape and completing the transformation into standard Prussian blue crystals, resulting in the PB50-24 sample. This process demonstrates the crucial role of water molecules in crystal growth and morphological transformation, as well as how extending the reaction time promotes the perfection of the crystal structure, leading to the formation of Prussian blue crystals low on defects.

**Figure 4:**
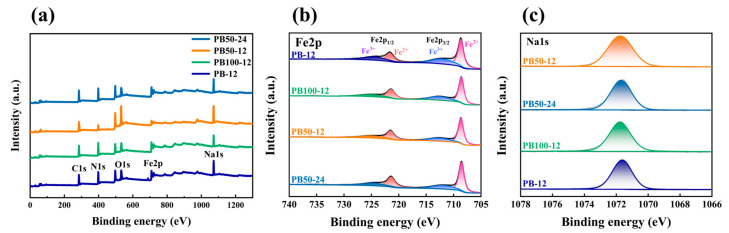
XPS spectra of (**a**) PB under different synthesis conditions, (**b**) the element Fe, and (**c**) the element Na.

To preliminarily analyze the oxidation level and low defect status of the samples, we conducted X-ray photoelectron spectroscopy (XPS) testing. Figure 4a presents the full-spectrum XPS patterns of various samples, revealing the presence of elements such as carbon (C1s), nitrogen (N1s), oxygen (O1s), iron (Fe2p), and sodium (Na1s). The high-resolution XPS spectrum focused on the iron (Fe) element, as shown in Figure 4b, displays the characteristic peaks of Fe^2+^ 2p_3/2_ and Fe^2+^ 2p_1/2_ at 708.5 eV and 721.3 eV, respectively, along with possible satellite peaks. The positions and intensities of these peaks help assess the oxidation state of iron in the samples, that is, the ratio of Fe^2+^ to Fe^3+^. The two satellite peaks at 711.24 eV and 723.28 eV are typically associated with the partial oxidation of Fe^2+^ to Fe³⁺ [29]; the smaller the area of these satellite peaks, the lower the degree of oxidation and potentially the higher the sodium content. The peak height of the Na1s XPS spectrum in Figure 4c roughly represents the sodium content on the sample surface, which is particularly important for evaluating the electrochemical performance of Prussian blue materials. Integrating these XPS data, it is evident that the sample synthesized at a 50% water concentration for 24 h has the lowest oxidation level of Fe^2+^ and the highest surface sodium content, indicating fewer defects generated during the crystal formation process. These analytical results are crucial for understanding the impact of different synthesis conditions on the surface chemical status of Prussian blue materials, especially their oxidation-reduction states and elemental composition, which are vital for comprehending the materials’ electrochemical performance.

To determine the sodium content in the samples, we conducted ICP testing, with the results presented in Table 1. The table lists the iron (Fe) and sodium (Na) content for three samples, PB-12, PB100-12, and PB50-24, measured in parts per million (ppm). The test results reveal the impact of synthesis time and water concentration on the sodium content in the samples—the PB50-24 sample, synthesized for 12 h at a 50% water concentration, exhibits the highest sodium content (91,795 ppm), while the PB100-12 sample, synthesized for 12 h at a 100% water concentration, shows the lowest sodium content (66,596 ppm). The sodium content of the PB-12 sample is 81,776 ppm. These data suggest that when the precursor is synthesized in an anhydrous state, the slower reaction rate results in fewer defects and thus a higher sodium content. In contrast, the sample at a 100% water concentration, due to the absence of a chelating agent, has a faster reaction rate, leading to more defects and consequently a lower sodium content. These differences may affect the electrochemical performance of the samples, with the high sodium content in the PB50-24 sample possibly indicating fewer defects generated during the crystal formation process.

**Figure 5:**
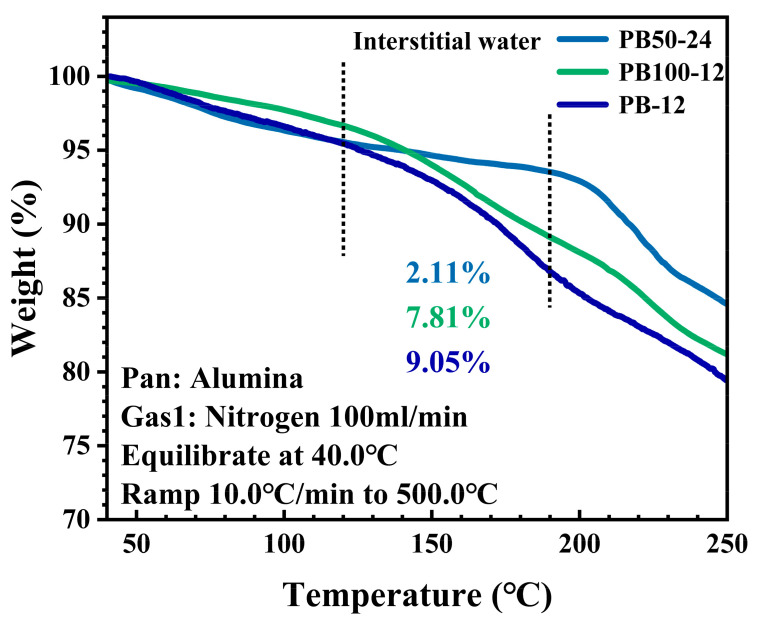
TGA curves of PB50-24, PB100-12, and PB-12.

Figure 5 presents a TGA of the water content in samples PB50-24, PB100-12, and PB-12, revealing the extent of defect formation within the samples. Under the testing conditions of 40 °C to 250 °C, all samples experienced weight loss, primarily due to the evaporation of adsorbed, interstitial, and coordinated water, while the weight loss between 120 °C and 190 °C is primarily attributed to the loss of interstitial water in the samples. Specifically, the weight losses for PB50-24, PB100-12, and PB-12 were 2.11%, 7.81%, and 9.05%, respectively. These results indicate that the samples synthesized by the new method (such as PB50-24) have a lower interstitial water content compared to those synthesized by traditional aqueous methods. This difference is attributed to the unique crystal growth characteristics of Prussian blue; even when chelating agents are added to slow down the reaction rate, some vacancies still occur during the crystal growth process, and water molecules do not fill these vacancies during the formation of the precursor. During the secondary synthesis, water molecules slowly enter the vacancies within the crystals to form a certain amount of coordinated water, facilitating the transformation of the crystals into standard Prussian blue crystals. This unique crystal formation mechanism results in relatively low interstitial water content in the crystals, leading to reduced weight loss during the TGA process. A lower water content also means that the crystal has fewer defects.

**Figure 6:**
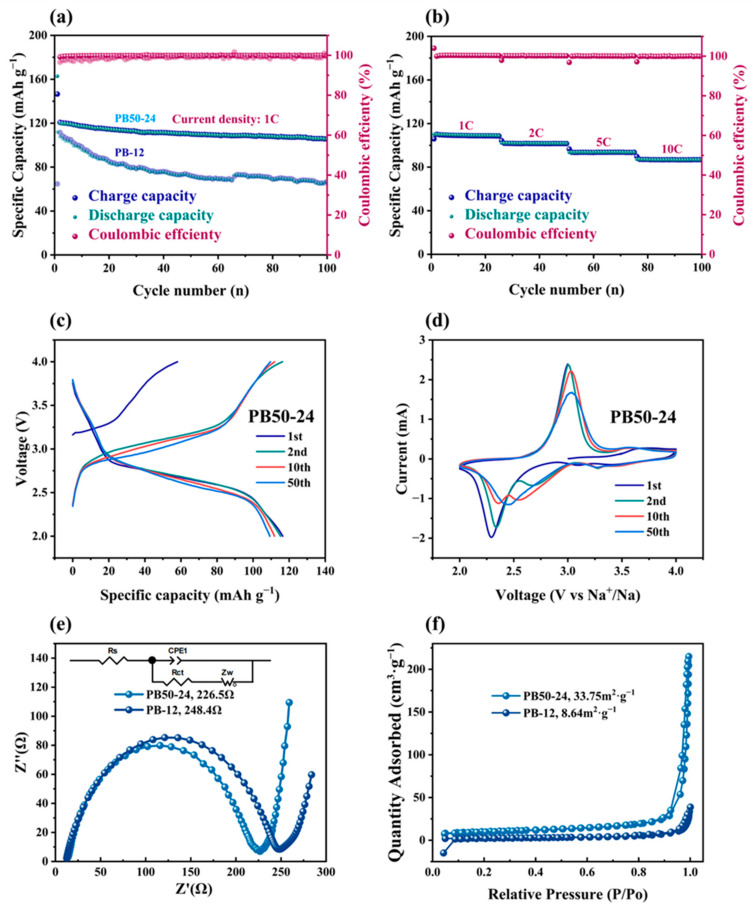
The electrochemical properties of the PB50-24 sample electrode. (**a**) Cycle life at 1C (compare with PB-12 sample), (**b**) charge and discharge capacity at different current densities, (**c**) charge–discharge profiles at 1C, (**d**) cyclic voltammetry (CV) curves, and (**e**) the EIS spectrum measured from 1 × 10^5^ to 0.01 Hz before the cycle. (**f**) N2 adsorption–desorption isotherms of the samples PB50-24 and PB-12.

Based on the material’s morphological characterization and elemental content analysis, the PB50-24 sample exhibits a more regular microstructure, with a higher sodium content and lower water content. Consequently, PB50-24 was selected as the active material to assemble a coin cell. Figure 6 illustrates the electrochemical performance of the PB50-24 sample electrode. Figure 6a shows the cycle life at a current density of 1C (170 mA g^−1^), where the battery demonstrates an initial discharge capacity of 120 mAh g^−1^ after activation, maintaining a capacity of 105 mAh g^−1^ after 100 charge–discharge cycles, indicating good cycling stability. The figure shows that PB50-24 has significantly better cycle stability compared to PB-12. Figure 6b presents the charge and discharge capacity at various current densities, from 1C to 10C, highlighting the battery’s excellent rate capability with a capacity of 86 mAh g^−1^ at a high current density of 10C. Figure 6c reveals the charge–discharge profiles at 1C, with the charging plateau at 3 V and the discharging plateau at 2.5 V, demonstrating the battery’s charge–discharge characteristics. The sample exhibits good discharge-specific capacity during the first charge–discharge cycle, and the capacity degradation is minimal during the second cycle. Figure 6d displays the cyclic voltammetry (CV) curves. During the first charging, Fe^2+^ undergoes an oxidation reaction, and sodium ions are de-intercalated. During the first discharge, Fe^3+^ undergoes a reduction reaction, and sodium ions are re-intercalated into the material, with the 1st, 2nd, 10th, and 50th cycle curves indicating that the sample has good reversibility. To further validate the reasons for its excellent electrochemical performance, electrochemical impedance spectroscopy, as shown in Figure 6e, and Nitrogen adsorption–desorption isotherms, as shown in Figure 6f, were conducted. The impedance of PB50-24 is 226.5 Ω, while the impedance of PB-12 is 248.4 Ω. PB50-24 has a lower internal resistance and a higher sodium-ion diffusion rate. The specific surface area of PB50-24 is also larger, at 33.75 m^2^ g^−1^, indicating more reaction sites, a more uniform material surface, and fewer defects. Therefore, PB50-24 exhibits better cycling charge and discharge capacity and good rate performance.

To compare the electrochemical performance of samples synthesized by the new synthesis method and the traditional aqueous synthesis method, and to explore the material’s application value, we compared several aqueous Prussian blue samples with excellent electrochemical performance in recent years, as shown in Table 2. PB50-24 still exhibits a high specific capacity at 170 mA g^−1^, which can be attributed to its good structure with fewer defects and lower water content. Under large currents, the sodium-ion migration barrier is relatively small, allowing for fast de-intercalation and re-intercalation, thus resulting in a high reversible specific capacity. These electrochemical performance comparison results indicate that the PB50-24 sample has potential application value in sodium-ion batteries, especially in applications requiring high stability and rate performance.

## 4. Conclusions

In summary, a novel synthesis approach for low-defect, low water content iron-based Prussian blue materials was successfully developed, which significantly enhanced the electrochemical performance of sodium-ion batteries. By synthesizing the precursor in a non-aqueous phase and carefully controlling the water concentration during secondary synthesis, we achieved Prussian blue crystals with optimized morphology, size, and reduced water content. The optimized material (PB50-24) demonstrated excellent electrochemical properties, including an initial discharge capacity of 120 mAh g⁻¹ at 1C, maintaining 105 mAh g⁻¹ after 100 cycles, and a rate capability of 86 mAh g⁻¹ at 10C. These results highlight the importance of controlling water concentration during synthesis to improve structural stability and electrochemical performance. This method not only provides a cost-effective and scalable route for synthesizing high-performance Prussian blue materials but also offers valuable insights for the development of other metal-based Prussian blue analogs. Future work will focus on further optimizing synthesis conditions and exploring the potential of these materials in large-scale energy storage systems.

## Figures and Tables

**Table 1:** ICP results for Fe and Na for PB-12, PB100-12, and PB50-24 samples.

Sample	Fe (ppm)	Na (ppm)
PB-12	219,940	81,776
PB100-12	297,016	66,596
PB50-24	203,450	91,795

**Table 2:** Comparison of electrochemical properties of NaFeHCF synthesized from other studies and our work.

Sample	Synthetic Medium	Specific Capacity (mAh g ^−1^)	Current Density (mA g ^−1^)
YSPB [33]	Water	118	170
BR-FeHCF [34]	Water	115	100
PB-S3 [35]	Water	99	100
HQ-NaFe [36]	Water	110	150
PB50-24	Glycerol, 50%Ethanol	120	170

## Data Availability

The original contributions presented in this study are included in the article. Further inquiries can be directed to the corresponding authors.
